# Characterizing poorly controlled type 2 diabetes using ^1^H-NMR metabolomics

**DOI:** 10.1007/s11306-024-02127-w

**Published:** 2024-05-11

**Authors:** Isabella J. Theron, Shayne Mason, Mari van Reenen, Zinandré Stander, Léanie Kleynhans, Katharina Ronacher, Du Toit Loots

**Affiliations:** 1 Human Metabolomics, Department of Biochemistry, Faculty of Natural and Agricultural Sciences, North-West University, Potchefstroom, South Africa; 2 DSI-NRF Centre of Excellence for Biomedical Tuberculosis Research, South African Medical Research Council Centre for Tuberculosis Research, Division of Molecular Biology and Human Genetics, Department of Biomedical Sciences, Stellenbosch University, Cape Town, South Africa; 3 Mater Research Institute, The University of Queensland, Translational Research Institute, Brisbane, Australia; 4 Australian Infectious Diseases Research Centre, The University of Queensland, Brisbane, Australia

**Keywords:** Poorly controlled type 2 diabetes, Metabolomics, Proton nuclear magnetic resonance, Urine

## Abstract

**Introduction:**

The prevalence of type 2 diabetes has surged to epidemic proportions and despite treatment administration/adherence, some individuals experience poorly controlled diabetes. While existing literature explores metabolic changes in type 2 diabetes, understanding metabolic derangement in poorly controlled cases remains limited.

**Objective:**

This investigation aimed to characterize the urine metabolome of poorly controlled type 2 diabetes in a South African cohort.

**Method:**

Using an untargeted proton nuclear magnetic resonance metabolomics approach, urine samples from 15 poorly controlled type 2 diabetes patients and 25 healthy controls were analyzed and statistically compared to identify differentiating metabolites.

**Results:**

The poorly controlled type 2 diabetes patients were characterized by elevated concentrations of various metabolites associated with changes to the macro-fuel pathways (including carbohydrate metabolism, ketogenesis, proteolysis, and the tricarboxylic acid cycle), autophagy and/or apoptosis, an uncontrolled diet, and kidney and liver damage.

**Conclusion:**

These results indicate that inhibited cellular glucose uptake in poorly controlled type 2 diabetes significantly affects energy-producing pathways, leading to apoptosis and/or autophagy, ultimately contributing to kidney and mild liver damage. The study also suggests poor dietary compliance as a cause of the patient’s uncontrolled glycemic state. Collectively these findings offer a first-time comprehensive overview of urine metabolic changes in poorly controlled type 2 diabetes and its association with secondary diseases, offering potential insights for more targeted treatment strategies to prevent disease progression, treatment efficacy, and diet/treatment compliance.

**Supplementary Information:**

The online version contains supplementary material available at 10.1007/s11306-024-02127-w.

## Introduction

Diabetes is a chronic, non-communicable, metabolic disorder, estimated to affect one in every 10 individuals globally (± 537 million individuals in 2021) (International Diabetes Federation, 2021). Diabetes encompasses a group of metabolic disorders characterized by dysregulation of cellular glucose uptake/metabolism, leading to impaired immune function and increased susceptibility to develop co-morbidities including cardiovascular diseases, nephropathy, neuropathy, coronavirus disease, tuberculosis, etc. (Geca et al., 2022; Klein & Shearer, 2016; Restrepo et al., 2018). If left untreated, the disease itself can rapidly progress, causing irreparable damage and even death. Consequently, diabetes ranks among the top ten leading causes of death globally, contributing to a mortality rate of ± 6.7 million diabetes patients in 2021 (International Diabetes Federation, 2021). While the implications of diabetes are similar, the exact pathophysiology and prevalence of the various subclassifications (i.e., type 1 and 2 diabetes, gestational diabetes, maturity-onset diabetes of the young, and neonatal diabetes) differ. Among these, type 1 and 2 diabetes are the most prevalent subtypes, with type 2 diabetes accounting for > 90% of all diabetes cases worldwide. It is caused by a combination of progressively worsening insulin resistance and the subsequent progressive decline of β-cell function over time (International Diabetes Federation, 2021). Type 2 diabetes can be subdivided into several phases based on disease progression ranging from insulin resistance to pre-type 2 diabetes, and ultimately type 2 diabetes (International Diabetes Federation, 2021). The American Diabetes Association (2020) standard diagnostic criteria are widely used to diagnose diabetic phases based on glycated hemoglobin (HbA1c), random blood glucose (RBG), and/or fasting blood glucose (FBG) levels. Pre-type 2 diabetes is classified as having an HbA1c of 5.7–6.4% and/or FBG of 5.6–6.9 mmol/L, while type 2 diabetes is typically diagnosed in patients with an HbA1c ≥ 6.5%, FBG ≥ 7 mmol/L, and/or RBG ≥ 11.1 mmol/L (American Diabetes Association, 2020). Maintaining good glycemic control is crucial to reduce the risk of developing complications and improve the overall well-being of type 2 diabetes patients. The guidelines provided by the American Diabetes Association (2015) advises that HbA1c levels should be maintained at < 7% to be considered well-controlled (American Diabetes Association, 2015). However, as the disease progresses, some patients struggle to control their HbA1c levels, even with the appropriate treatment, resulting in an increased prevalence of poorly controlled type 2 diabetes (Bencharit et al., 2022). Unfortunately, literature on poorly controlled type 2 diabetes is inconsistent, since different cut-off values are used that range from HbA1C ≥ 7% with a pre-prandial capillary plasma glucose of ≥ 7.2 mmol/L (Arosemena Coronel et al., 2015) to HbA1C ≥ 9% with an FBG of ≥ 12 mmol/L (Swetha, 2014). Despite the well-characterized physiological and immunological impacts of type 2 diabetes, researchers have aimed at utilizing metabolomics to identify specific metabolites that play a role in characterizing these occurrences. Metabolomics allows for the identification and quantification of metabolic changes resulting from specific perturbations in chemical processes and mechanisms (Klein & Shearer, 2016). Since the metabolome is the downstream product of the genome, transcriptome, and proteome, it is the closest to the phenotype of the biological system. Consequently, it provides in-depth information on the altered physiological state of type 2 diabetes (Klein & Shearer, 2016). In addition to elevations in blood glucose concentrations, previous metabolomic investigations have observed aberrant metabolic fluctuations of other carbohydrate metabolites, including myo-inositol, fructose, and mannose, due to insulin resistance in patients with type 2 diabetes (Ahola-Olli et al., 2019; Ferrannini et al., 2020; Xu et al., 2013; Yang et al., 2018). Furthermore, an increase in fatty acids, ketone bodies, and amino acids (AAs) was also observed, which supports the metabolic flexibility of these patients to use alternative fuel substrates (Ahola-Olli et al., 2019; Salway, 2017; Xu et al., 2013). This adaptability arises from impaired glucose uptake/metabolism and is accompanied by a redox imbalance induced by the decline in glycolysis and the concurrent increase in fatty acid oxidation (Wu, 2016). While these studies provide a relatively comprehensive overview of the metabolic pathways affected by type 2 diabetes, limited literature is aimed at characterizing the metabolic changes associated with poorly controlled type 2 diabetes. In one of the few existing studies, Yun et al. (2019) showed significantly different blood glucose levels when comparing type 2 diabetes patients with an HbA1C ≤ 6% to those with HbA1C levels ≥ 9%, using liquid chromatography and flow-injection analysis coupled with mass spectrometry. Furthermore, the study observed a decrease in certain amino acids (glutamine, tryptophan, histidine, lysine, valine, glycine, serine, sarcosine, threonine, tyrosine) and two phosphatidylcholines (lysoPC a C16:1 and lysoPC a C18:0), while six other phosphatidylcholine metabolites (PC aa C34:1, PC aa C36:1, PC aa C28:1, PC C36:4, PC aa C26:0, and PC aa C34:2) were elevated in patients with higher levels of HbA1C (Yun et al., 2019). Additionally, this study suggested that certain metabolites such as glycine, valine, and phosphatidylcholines could be directly associated with an increase in HbA1c levels. On the contrary, Taya et al. (2021) and Alqudah et al. (2021) identified discrepancies in the direction and magnitude of the metabolic flux of the branched chain AA (BCAA; leucine, valine, isoleucine), aromatic AA (AAA; tryptophan and phenylalanine) and lysine’s concentrations. However, this variation can be attributed to external factors such as response to treatment (Alqudah et al., 2021; Taya et al., 2021), exercise and dietary intake (Alqudah et al., 2021). Although these studies provide credible information, limited literature is available on urine metabolic changes in poorly controlled type 2 diabetes patients. Using urine is advantageous because of its inherent sterility, abundant availability, non-invasiveness, and generally high compliance during collection (Zhang et al., 2012). Considering this, an untargeted ^1^H-NMR metabolomics approach was used to holistically compare the urine metabolite profiles of poorly controlled type 2 diabetes patients (*n* = 15) to those of healthy controls (*n* = 25). Thus, this study aimed to better characterize and understand the metabolic occurrences of poorly controlled type 2 diabetes.

## Methods and materials

This investigation forms part of the larger ALERT study, which consists of multiple interdisciplinary aims. Hence, additional information pertaining to the larger cohort participant recruitment, selection, auxiliary clinical participant information, and physiological measurements are described in detail by Restrepo et al. (2018), while only the information specific to the smaller cohort subset utilized in this metabolomics investigation is described below.

### Participants

Study participants (*n* = 40; *n* = 25 healthy controls and *n* = 15 poorly controlled type 2 diabetes patients) were recruited as part of the ALERT study from community clinics in the Northern suburbs of Cape Town (Western Cape, South Africa) (Restrepo et al., 2018). Participation in this investigation was completely voluntary and prior to any sampling procedures related to the larger study, the participants provided written and informed consent. The initial eligibility assessment involved sociodemographic and health questionnaires (age, sex, smoking, and medical history). Both males and females between the ages of 30 and 65 years, with a body-mass index > 20 kg/m^2^, were included (Restrepo et al., 2018). Type 2 diabetes was classified based on HbA1c ≥ 6.5% and FBG ≥ 11.1 mmol/L and included both newly diagnosed and known diabetes patients. Participants who were pregnant, had TB/HIV, other infections, cancer or used illicit drugs and/or excessive alcohol were excluded (Restrepo et al., 2018). For the current metabolomics study, further stratification was performed, incorporating healthy controls with HbA1c levels ≤ 5.7% and/or average FBG levels ≤ 5.6 mmol/L, as well as including poorly controlled type 2 diabetes participants with HbA1c levels ≥ 9%, and/or average FBG levels ≥ 12 mmol/L (Swetha, 2014). Sociodemographic information for these participants is provided in Table 1, while relevant treatment regimens can be obtained in S1 Table 1. Ethical clearance for the ALERT study was granted by the Stellenbosch University Health Research Ethics Committee (N13/05/064A), while approval for the metabolomics investigation was obtained from the North-West University Health Research Ethics Committee (ethics number: NWU-00336-21-S1), both in accordance with the Declaration of Helsinki.

**Table 1:** Sociodemographic characteristics of healthy control and poorly controlled type 2 diabetes participants

Participant characteristics	Healthy control (*n* = 25)	Poorly controlled type 2 diabetes (*n* = 15)
**Age (years) (mean ± standard deviation**)	43.78 ± 7.91	54.80 ± 4.81
**Gender (males; females)**	5; 20	1; 14
**Smoking (%)**		
Never smoked	32	13
Past smokers	0	27
Current smokers	68	60
**BMI (Kg/m**^**2**^**) (mean ± standard deviation**)	24.82 ± 5.29	28.45 ± 5.98
**HbA1c (%) (mean ± standard deviation**)	5.22 ± 0.29	11.55 ± 1.19
**FBG (mmol/l) (mean ± standard deviation**)	4.55 ± 0.67	14.87 ± 2.68

HbA1c, glycated haemoglobin; FBG, fasting blood glucose

### Urine sample collection and storage

Urine samples (2–5 mL mid-stream) from all participants were collected in standard de-identified urine collection vials, after ± 8 h of fasting. The samples were stored at -80 °C in the Stellenbosch University biobank. The samples were then transported to the North-West University in temperature-controlled transport boxes, where all samples remained stored in -80 °C bio-freezers until metabolomics analyses commenced.

### Urine buffer preparation

The urine buffer solution, used to maintain a constant pH (7.4) and lock the signal during ^1^H-NMR analysis, was prepared in advance, as described by Bester (2021). Briefly, 1.5 M monobasic potassium phosphate (KH_2_PO_4_) buffer solution was prepared by dissolving 20.4 g of it in 80 mL of deuterium oxide (D_2_O). Next, 13 mg of sodium azide (NaN_3_; anti-microbial agent) and 100 mg of trimethylsilyl-2,2,3,3-tetradeuteropropionic acid (TSP; internal standard) were dissolved in 8 mL of D_2_O. These solutions were then combined and vortexed. Next, potassium hydroxide (KOH) pellets were added to adjust the pH to 7.4. The combined solution was transferred to a volumetric flask and diluted to reach a final volume of 100 mL with D_2_O.

### Urine sample preparation

Before sample preparation, a pooled quality control (QC) sample was prepared by combining 75 µL from each sample and dividing it into three separate aliquots to be analyzed with each batch. Hereafter, all samples were prepared as described by Davoren (2023). Briefly, 1000 µL from each of the samples and QC aliquots were centrifuged at 12 000 x *g* for 5 min, before transferring 540 µL supernatant to a microcentrifuge tube. Next, 60 µL of the urine buffer solution (90% sample: 10% buffer) was added to the samples and vortexed. These were then centrifuged at 12 000 x *g* for 5 min, before transferring 540 µL supernatant to a 5 mm NMR glass tube and capping it.

### ^1^H-NMR analysis

The ^1^H-NMR method and data management, used in this investigation, have been extensively described in both Davoren (2023) and Bester (2021). In short, all prepared healthy controls and poorly controlled type 2 diabetes samples were randomized and divided into three separate batches, and QC samples were placed at the beginning, middle, and end of each batch. These samples were then analyzed on a Bruker Avance III HD 500 MHz NMR spectrometer. The following steps were then performed on Topspin (V3.5) to adjust the experimental parameters for each sample analysis: (**1)** The ^1^H-NMR was locked onto the deuterated components in the sample, allowing a homogeneous magnetic field. **(2)** Automatic shimming of the sample was performed on the TSP signal, aligning the applied magnetic field during the analyses. **(3)** The probe was tuned to 500.133 MHz. **(4)** Lastly, Nuclear Overhauser Effect Spectroscopy (NOESY-presat) was applied, to ensure water suppression and a pulse angle of 90° for each sample for 8 µs followed by a 4 s relaxation delay (Bester, 2021).

### Data management

Data pre-processing steps were performed using Bruker Topspin (V3.5) software. In summary, the raw data underwent a Fourier transformation, baseline phasing, and automated data correction. The TSP peak was then calibrated to 0.00 ppm (Davoren, 2023). The quality of the spectral resolution was manually checked by measuring the TSP peak at half-height in width (< 1 Hz) and ensuring that there were two small satellite peaks; one on each side of the TSP peak (Davoren, 2023). Furthermore, a verification process was conducted to ensure effective suppression of the water resonance peak at approximately 4.72 ppm. Data processing was performed by utilizing Bruker AMIX (V3.9.14) software. To account for inter-patient urine dilution, all samples were normalized relative to the creatinine peak signal at 4,05 ppm (Davoren, 2023). Furthermore, spectral binning was performed by segmenting the spectra into set-width bins of 0.02 ppm in size. Data clean-up was performed in Microsoft Excel (2019) and involved identifying bin regions without peaks (blanks) in all comparative group spectra. Hereafter, the limit of detection (LOD) was estimated to be 0.001 mmol, using the average of the blank bins and adding 3.3 times the standard deviation (SD) of the blank bins (LOD = MeanBlank + 3.3(SD Blank) (Westgard, 2008). All integral bin values below the LOD were removed (left blank), while the remaining bin dataset was imported into Metaboanalyst (V5.0). The data was then subjected to 50% zero/missing value filtering. The remaining missing values were replaced by a fifth of the minimum value of the entire dataset, followed by a QC CV filter to remove bins with a CV > 40%. Lastly, the data were log-transformed and Pareto scaled (multivariate analysis only) before being subjected to a multi-statistical approach.

### Statistical analysis

**Fig. 1:**
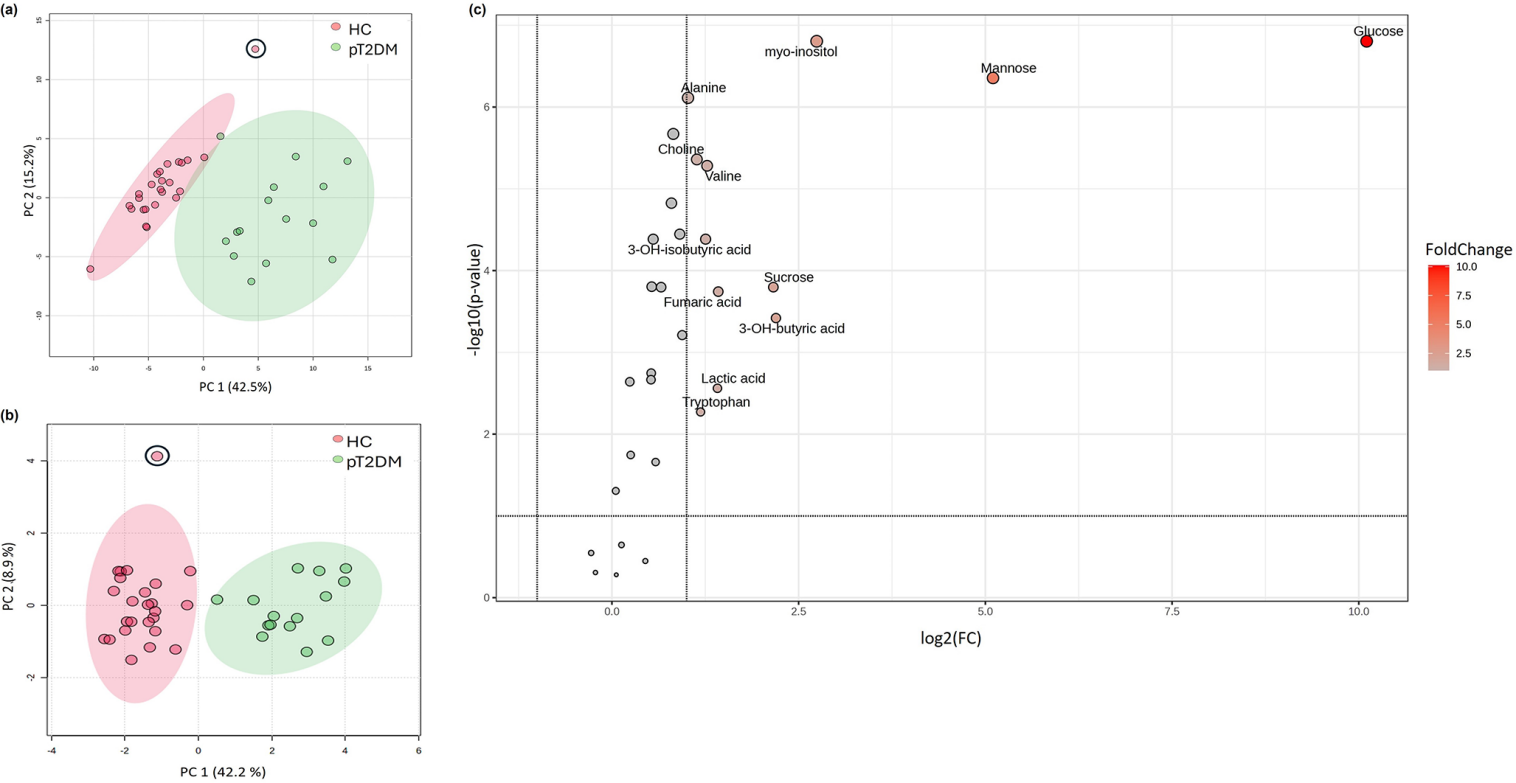
Bin and metabolite PCA results and a volcano plot. **(a)** The PCA score plot illustrates a natural differentiation in the bin data of the urine metabolome profiles of the 25 healthy control (HC) (pink circle) and 15 poorly controlled type 2 diabetes (pT2D) (green circle) participants. **(b)** The PCA score plot illustrates natural differentiation in the 25 metabolites of the urine metabolome profiles of the 25 HC (pink circle) and 15 pT2D (green circle) participants. **(c)** The volcano plot visually depicts metabolites based on their combined biological and statistical significance. Non-parametric FDR-adjusted p-values, log transformed with base 10, are depicted on the y-axis, with a significance threshold of 0.1, making no assumption on the equality of group variances. The x-axis represents log2 scaled fold change (FC) values considered practically relevant if |FC| ≥ 2.0 when comparing metabolite profiles of the poorly controlled type 2 diabetes cohort relative to the healthy control cohort. *The circled participant has been classified as an outlier

To visualize the metabolic differentiation between the comparative groups, the bin data were subjected to multivariate statistical methods, including an unsupervised principal component analysis (PCA) and a supervised partial least squares discriminant analysis (PLS-DA), both performed using Metaboanalyst (V5.0) (van Zyl et al., 2020). Bin/metabolite selection relied on univariate statistical methods, including a non-parametric Wilcoxon rank-sum test (adjusted for multiple tests using the Benjamini-Hodgeberg approach (Jafari & Ansari-Pour, 2019) to control the false discovery rate (FDR) (Metaboanalyst (V5.0), as well as a Glass’s ∆ effect size (Excel 2019) (Ialongo, 2016). These statistical selection methods were implemented in two phases, as described in detail by Bester (2021). In phase one, all statistically significant bins were selected using a large effect size (d-value) ≥ 0.8 and an FDR-adjusted p-value ≤ 0.05. Hereafter, the peaks within these statistically significant bins were identified and quantified, as described in the following section. In phase two, discriminatory metabolites were selected using a moderate effect size (d-value) ≥ 0.5 and an FDR-adjusted p-value ≤ 0.05. S1 Fig. 1 summarizes the multi-statistical approach used in this study.

### Metabolite identification and quantification

Metabolites were identified using a pure chemical compound spectral library. The ^1^H-NMR assignments are stipulated in S1 Table 2. The identified metabolites were quantified relative to creatinine, in Excel (2019), as described by Davoren (2023). In short, this process involved: (1) calculating the quotient of the peak integral and the number of protons in each metabolite; (2) dividing the signal area per proton of the identified compound by the signal area per proton of creatinine, multiplied by 1000 (mmol/mol creatinine). Subsequently, an additional noise removal step was performed that included manual verification of the spectra of each participant in Bruker AMIX (V3.9.14), was performed on the quantified data.

## Results

**Fig. 2:**
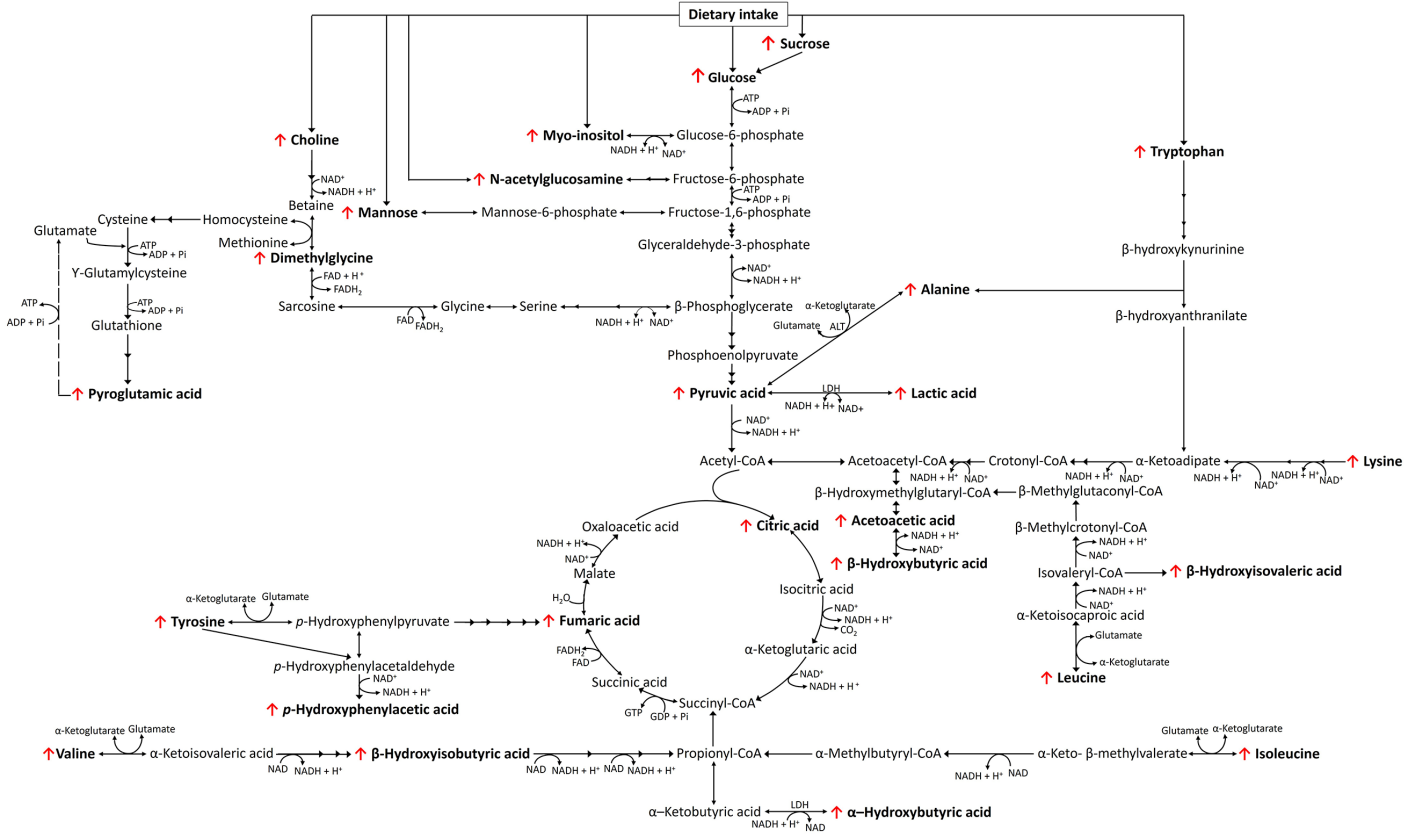
A schematic summary of the metabolic changes associated with poorly controlled type 2 diabetes in this metabolomics investigation. The directional changes in the concentrations of the metabolite markers are shown to increase (↑) or decrease (↓) relative to the healthy control group. FAD flavin adenine dinucleotide, FADH flavin adenine dinucleotide + hydrogen, NAD nicotinamide adenine dinucleotide, NADH nicotinamide adenine dinucleotide + hydrogen, ATP adenosine triphosphate, ADP adenosine diphosphate

The PCAs revealed a distinct separation between the metabolic profiles of healthy control and poorly controlled type 2 diabetes patients. Based on the 95% confidence intervals for the score centroids, one outlier was observed in the healthy control group (Fig. 1a, and b). After re-evaluating the participant’s clinical data, analytical data, and participant characteristics, no justification for the removal of the participant could be made. Importantly, the presence of this outlier did not significantly influence the group clustering or differentiation between the comparative groups. Consequently, the participant was retained in the analysis. The PLSDA (S1 Fig. 2.a) indicated a separation between the comparative groups and was further validated using various methods (S1 Fig. 2b and c), thus confirming the reliability of the multivariate model. To identify the metabolites that significantly contribute to the group separation observed in S1 Fig. 1, a two-step multi-statistical approach was employed as described in the statistical analysis section and S1 Fig. 1. During phase one, 144 statistically significant spectral bins (from the initial 455 bins) were identified and subsequently quantified. Hereafter, phase two of the statistical analysis commenced, yielding a total of 25 interpretable metabolite markers. A volcano plot visually depicts the metabolites according to their statistical significance (Fig. 1c). In particular, glucose emerged as the most statistically significant metabolite observed in the poorly controlled type 2 diabetes cohort compared to the healthy control cohort, aligning with expectations. Additionally, Table 2 provides a summary of the average concentrations, standard deviations, and d- and p-values, which were used for the variable selection of the statistically significant metabolites.

**Table 2:** Statistically significant urine metabolites that describe the variance in the metabolome between the healthy control and poorly controlled type 2 diabetes cohorts

Metabolites	Healthy control	Poorly controlled type 2 diabetes	Healthy control vs. Poorly controlled type 2 diabetes	
	Average concentration ± Standard deviation (mmol/mol creatinine)		Glass’s σ effect size (≥ 0.5)	FDR-adjusted *p*-value (≤ 0.05)
**α-Hydroxybutyric acid** *(HMDB0000008)*	1.45 ± 0.14	1.68 ± 0.33	1.61	1.33 × 10^− 3^
**β-Hydroxybutyric acid** *(HMDB0000011)*	0.46 ± 0.45	1.20 ± 0.49	1.65	3.94 × 10^− 4^
**β-Hydroxyisobutyric acid** *(HMDB0000023)*	1.10 ± 0.29	1.51 ± 0.19	1.38	4.27 × 10^− 5^
**β-Hydroxyisovaleric acid** (*HMDB0000754)*	1.13 ± 0.12	1.28 ± 0.14	1.31	2.71 × 10^− 3^
**ρ-Hydroxyphenylacetate** *(HMDB0060390)*	1.32 ± 0.31	1.55 ± 0.19	0.71	2.17 × 10^− 2^
**Acetoacetic acid** *(HMDB0000060)*	1.45 ± 0.11	1.70 ± 0.18	2.32	3.71 × 10^− 5^
**Alanine** *(HMDB0000161)*	1.68 ± 0.12	1.98 ± 0.14	2.57	7.98 × 10^− 7^
**Choline** *(HMDB0000097)*	0.93 ± 0.37	1.35 ± 0.13	1.15	4.52 × 10^− 6^
**Citric acid** *(HMDB0000094)*	2.35 ± 0.38	2.71 ± 0.17	0.95	6.35 × 10^− 4^
**Dimethylglycine** *(HMDB0000092)*	1.00 ± 0.16	1.18 ± 0.09	1.15	1.63 × 10^− 4^
**Fumaric acid** *(HMDB0000134)*	3 × 10^− 3^ ± 0.48	0.55 ± 0.24	1.14	2.00 × 10^− 4^
**Glucose** *(HMDB0000122)*	0.92 ± 0.21	3.81 ± 0.57	13.75	1.62 × 10^− 7^
**Isoleucine** *(HMDB0000172)*	0.69 ± 0.08	0.85 ± 0.10	2.01	4.27 × 10^− 5^
**Lactic acid** *(HMDB0000190)*	1.81 ± 0.37	2.20 ± 0.32	1.05	2.71 × 10^− 3^
**Leucine** *(HMDB0000687)*	1.37 ± 0.19	1.63 ± 0.12	1.36	2.20 × 10^− 6^
**Lysine** *(HMDB0000182)*	2.10 ± 0.23	2.25 ± 0.26	0.64	1.75 × 10^− 3^
**Mannose** *(HMDB0000169)*	0.18 ± 0.24	1.56 ± 0.52	5.70	4.56 × 10^− 7^
**Myo- inositol** *(HMDB0000211)*	< 0.001	2.37 ± 0.28	1.4 × 10^15^	1.62 × 10^− 7^
**N-acetylglucosamine** *(HMDB0000215)*	1.72 ± 0.07	1.80 ± 0.05	1.06	2.37 × 10^− 3^
**Pyroglutamate** *(HMDB0000267)*	2.44 ± 0.10	2.51 ± 0.10	0.74	1.78 × 10^− 2^
**Pyruvic acid** *(HMDB0000243)*	1.11 ± 0.19	1.33 ± 0.18	1.17	3.75 × 10^− 4^
**Sucrose** *(HMDB0000258)*	1.15 ± 0.43	1.77 ± 0.54	1.50	1.78 × 10^− 4^
**Tryptophan** *(HMDB0000929)*	1.00 ± 0.53	1.50 ± 0.44	0.95	5.29 × 10^− 3^
**Tyrosine** (*HMDB0000158)*	1.10 ± 0.33	1.41 ± 0.08	0.95	1.55 × 10^− 5^
**Valine** *(HMDB0000883)*	0.64 ± 0.40	1.16 ± 0.10	1.29	5.41 × 10^− 6^

HMDB, The human metabolomics database

## Discussion

Table 2 summarizes the metabolites that best describe the differences in urinary metabolome between the healthy control and poorly controlled type 2 diabetes cohorts. It mainly includes intermediates associated with macro fuel substrate pathways, namely carbohydrates, ketogenesis, proteolysis, and the tricarboxylic acid (TCA) cycle, primarily induced by the inhibition of glucose utilization/cellular uptake in the poorly controlled type 2 diabetes patients. These metabolite pathways are comprehensively discussed in the following and are presented schematically in Fig. 2.

### Carbohydrate metabolism

Given that insulin resistance and β-cell dysfunction are established factors that result in compromised cellular glucose uptake (via inhibition of GLUT4) and impaired carbohydrate catabolism (via diminished glucokinase activity) in individuals with type 2 diabetes (Ferrannini et al., 2020; Salway, 2017), it is expected to see elevated concentrations of urinary glucose and metabolites associated with gluconeogenesis, such as mannose (Ferrannini et al., 2020), in the poorly controlled type 2 diabetes cohort. Furthermore, there were notable elevations in myo-inositol concentrations, which further confirmed elevated gluconeogenesis in poorly controlled type 2 diabetes patients. This observation can be attributed to glucose-induced competitive inhibition of sodium-dependent myo-inositol absorption in the renal system, a phenomenon associated with the structural similarities shared by myo-inositol and glucose (Croze & Soulage, 2013). Moreover, according to Park et al. (2018), the observed elevation in urinary myo-inositol in individuals with type 2 diabetes may serve as a biological marker indicating non-responsiveness to metformin treatment. In addition to the pathophysiological mechanisms resulting in the elevated concentrations of the aforementioned metabolic intermediates, an increased urinary sucrose excretion (a known dietary marker for elevated sucrose intake) observed in this cohort, indicates a lack of compliance of the poorly controlled type 2 diabetes cohort with the dietary requirements generally prescribed for type 2 diabetes patients (Tasevska, 2015).

### TCA cycle and ketogenesis

The inability of type 2 diabetes patients to utilize glucose efficiently and the resulting impaired glycolysis, leads to the catabolism of alternative fuel substrates, such as lipids and proteins, and the subsequent increased influx of acetyl-CoA and propionyl-CoA into the TCA cycle (Fig. 2) (Salway, 2017, Wu, 2016). Subsequently, this leads to upregulation of TCA cycle enzyme activity, supported by elevated concentrations of both citric acid and fumaric acid concentrations in the poorly controlled type 2 diabetes cohort. Consequently, these energy-dependent metabolic changes and the resulting accumulation of nicotinamide adenine dinucleotide and hydrogen (NADH) and flavin adenine dinucleotide (FADH_2_) have been suggested to partially saturate the electron transport chain (ETC), resulting in the imbalanced redox potential (NAD^+^: NADH and FAD: FADH_2_) commonly observed in patients with type 2 diabetes (Salway, 2017, Wu, 2016). An imbalanced redox potential is hypothesized to influence several NAD^+^ and NADH-dependent dehydrogenase enzymes involved in carbohydrate, lipid, and protein metabolism (Fig. 2). In this investigation, increased concentrations of pyruvic acid in poorly controlled type 2 diabetes patients hampering the action of pyruvic acid dehydrogenase supports this occurrence. According to Sas et al. (2016), elevated pyruvic acid could also be an indication of an increase in flux through the glycolysis pathway in the kidneys. Moreover, this study further suggested that this phenomenon, along with the increased flux through the TCA cycle, as seen in this poorly controlled type 2 diabetes cohort, has typically been associated with kidney disease (Sas et al., 2016). In an attempt to circumvent this imbalanced redox potential, the accumulated pyruvic acid is converted to lactic acid via lactic acid dehydrogenase, which uses NADH to regenerate NAD^+^ (Wu, 2016), thus further supporting the observed increase in lactic acid within the poorly controlled type 2 diabetes patient cohort. Additionally, external factors such as metformin treatment may further contribute to increased concentrations of lactic acid and ketone bodies (β-hydroxybutyric acid and acetoacetate), via the inhibition of mitochondrial respiration (DeFronzo et al., 2016, Schwetz, 2017), also evident in our investigation. Increased ketone bodies can also be attributed to the continuous influx of acetyl-CoA from the aforementioned metabolism of alternative energy producing substrates (lipids and proteins), and an imbalanced redox potential, which promotes ketogenesis (another NAD^+^ regeneration pathway) (Fig. 2). The latter is not surprising, as ketone bodies are an important alternative fuel source for the brain, kidney, and skeletal muscles, and are able to freely diffuse across the cell membranes of these organs to sustain functionality under conditions of inhibited glucose availability (Puchalska & Crawford, 2017; Salway, 2017). Interestingly, in this cohort of poorly controlled type 2 diabetes patients, slightly higher concentrations of acetoacetic acid compared to β-hydroxybutyric acid were observed. According to Noyes et al. (2007), this may occur in patients with diabetic ketoacidosis (DKA) who received insulin treatment. The latter is suggested to reduce β-hydroxybutyric acid concentrations long before acetoacetic acid concentrations change, due to the stimulating effect of insulin treatment on the β-hydroxybutyric acid dehydrogenase enzyme (Noyes et al., 2007). This phenomenon, along with elevations in lactic acid (Bhat et al., 2021) and ketonuria (Brooke et al., 2016) are commonly observed in type 2 diabetes and has been associated with DKA (Salway, 2017).

### Proteolysis

Elevated concentrations of several amino acids (alanine, BCAA, lysine, tyrosine, and tryptophan) and their associated catabolism intermediates (β-hydroxyisobutyric acid, β-hydroxyisovaleric acid, p-hydroxyphenylacetic acid, and α-hydroxybutyric acid) were also observed in the poorly controlled type 2 diabetes patients. These findings align with existing literature, indicating the use of dietary and/or endogenous proteins as alternative fuel substrates in type 2 diabetes patients (Chen et al., 2016; Xu et al., 2013). Notably, the synthesis of most of these intermediates also relies on various NADH/NAD^+^ dependent dehydrogenase enzymes (Fig. 2) and may subsequently also be ascribed to the hampering of these enzymes due to an imbalanced redox potential in these participants. Elevated excretions in BCAA, particularly leucine, have also been proposed to overstimulate the mammalian target of rapamycin complex 1 (mTORc1) in the early stages of type 2 diabetes (Melnik, 2012). Short-term activation of mTORc1 serves as a compensatory mechanism to increase insulin secretion by promoting β-cell growth and proliferation. However, as type 2 diabetes progresses, an overload of nutrients results in chronic hyperactivation of mTORc1 (Melnik, 2012). A sustained hyperactivation of mTORc1 in time results in mitochondrial dysfunction, insulin resistance, and ultimately a depletion of β-cell mass via apoptosis (PCD; programmed form of cell death).

(Melnik, 2012, Yarahmadi et al., 2021). Furthermore, research has indicated that oxidative stress also triggers apoptosis in pancreatic β-cells (Zhao & Wang, 2012), which is supported by the elevated excretion of pyroglutamic acid (oxidative stress marker) in the poorly controlled type 2 diabetes cohort in our study (van der Pol et al., 2018). Apoptosis is responsible for degrading/removing damaged cells via phagocytosis (Westman et al., 2019), however, in diabetes patients, clearance of damaged β-cells via this mechanism is thought to decline as the disease progresses, leading to partially digested apoptotic bodies. The latter then progressively results in a loss of membrane potential, and the subsequent formation of secondary necrotic cells, leading to the leakage of cellular constituents (Kim & Lee, 2010; Sachet et al., 2017), explaining the observed increase in proteins and other cell membrane constituents (Sachet et al., 2017), such as phospholipid derivatives (choline, myo-inositol (Chaurio et al., 2009) and glycan structural components (mannose, N-acetylglucosamine (Rapoport & Pendu, 1999) in these poorly controlled type 2 diabetes patients. In contrast to this, several studies have also reported hyperactivation of autophagy in type 2 diabetes patients, as a compensatory mechanism to recycle various cellular contents for the production of adenosine triphosphate during hypoglycemic/fasting states (Denton & Kumar, 2019; Mohammadi-Motlagh et al., 2023; Yang et al., 2017). Interestingly, it has also been suggested that the increased autophagy that occurs in diabetes patients β-cells may also be caused by metformin treatment (Jiang et al., 2014), and a secondary inhibition of mTORc1 (Blandino-Rosano et al., 2017). Chronic hyperactivation of autophagy in diabetes patients also results in an elevated number and size of autophagic vacuoles in β-cells, which in turn can also further trigger autophagic cell death or autophagic-mediated cell death mechanisms in the pancreas (Denton & Kumar, 2019; Yang et al., 2017). Hence, it is plausible that both apoptosis and autophagy play a role in the cell membrane constituents observed in these poorly controlled type 2 diabetes patients, which warrants further investigation of these mechanisms. However, these PCD mechanisms have been associated with an increased risk of diabetes complications, as they contribute to cellular dysfunction and damage in various tissues, such as the kidney and liver, as the disease progresses (Bhattacharya et al., 2018; Yarahmadi et al., 2021). This is supported by the observed increases in metabolites that have been associated with kidney damage (myo-inositol (Chang et al., 2015), dimethylglycine (McGregor et al., 2001), citric acid (Sas et al., 2016), and pyroglutamic acid (Mutter et al., 2022) and mild liver damage (p-hydroxyphenylacetic acid (Ghoraba et al., 2014) within this poorly controlled type 2 diabetes cohort.

## Conclusion

The current investigation provides a holistic overview of the urinary metabolic adaptations induced by poorly controlled type 2 diabetes in a South African cohort. The findings revealed significant strain on alternative energy-producing substrate pathways such as carbohydrates, ketogenesis, and protein catabolism, to sustain biological functionality. Consequently, the influence of these pathways on the TCA cycle and ETC results in diabetic ketoacidosis and an imbalanced redox potential, which in turn hampered several redox-dependent enzymes involved in amino acid and lipid metabolism. In addition to these changes to the traditional catabolic pathways, this investigation identified metabolic patterns indicative of autophagy and/or apoptosis as additional compensatory mechanisms for the dysregulation of cellular glucose uptake/metabolism in these poorly controlled type 2 diabetes patients. Activation of these PCD mechanisms also supported the detection of elevated concentrations of various metabolites associated with kidney and mild liver damage, further supporting the complexity of the disease state. These kidney and liver damage markers are of particular interest, as they can be used to determine not only disease severity, but also early-stage damage during a phase of kidney and liver stress before standard kidney and liver markers are abnormal and can be subsequently used for preventative treatment strategies. Additionally, the study indicates that poor dietary compliance is one of the causes of the uncontrolled glycemic state of these patients, and the monitoring of sucrose could potentially serve as a marker for monitoring this. While acknowledging inevitable limitations such as participant genotype/phenotype variation, and uncontrolled dietary intake, the study design pertinently excluded participants with active TB, HIV, or any other infections, as stated by Restrepo et al. (2018). This, in conjunction with the stringent selection of patients with a HbA1c of ≥ 9% and FBG ≥ 12 mmol/L, led to a reduction in the size of the patient cohorts, within the current investigation. Considering this, the true value of the study is in its ability to inform follow-up research and not in its generalizability. Subsequently, future investigations consisting of larger cohorts originating from different geographic locations may serve to not only substantiate the validity of these findings, but also provide insights to alternative informative metabolic pathways/metabolites and potential metabolic differences between genders. Finally, the metabolic patterns observed in poorly controlled type 2 diabetes indicate both apoptosis and/or autophagy as additional compensatory mechanisms for the reduced uptake of glucose intracellularly in patients suffering from this severe disease state. However, the exact role and interaction of these mechanisms in type 2 diabetes remain controversial and require further investigation. Hence, future investigations should aim to elucidate the precise nature of these mechanisms in type 2 diabetes patients to provide possible targets for more efficient treatment/management options. In conclusion, the results of this study not only provide valuable information on the pathophysiological mechanism of poorly controlled type 2 diabetes and the consequential secondary disease states induced by this condition, but also provide clues towards the development of more targeted interventions aimed at preventing the progression of type 2 diabetes to a poorly controlled diabetic state. Finally, the study also presents potential markers to monitor diabetes progression and assess adherence to treatment and dietary compliance.

### Electronic supplementary material

Below is the link to the electronic supplementary material.

Supplementary Material 1

## Data Availability

The current investigation forms part of the broader ALERT study that consists of multiple interdisciplinary aims, which are being drafted into various manuscripts. Considering this, the raw data supporting the findings of this manuscript can be acquired from the corresponding author upon reasonable request.

## Abbreviations

HbA1c: Glycated hemoglobin
FBG: Fasting blood glucose
RBG: Random blood glucose
^1^H-NMR: Proton nuclear magnetic resonance
AA: Amino acid
BCAA: Branched chain amino acid
AAA: Aromatic amino acid
TCA: Tricarboxylic acid cycle
NADH: Nicotinamide adenine dinucleotide and hydrogen
FADH_2_: Flavin adenine dinucleotide
ETC: Electron transport chain
GLUT4: Glucose transporter type 4
mTORc1: Mammalian target of rapamycin complex 1
