# Supplementary material for: Characterizing poorly controlled type 2 diabetes using 1H-NMR metabolomics
Source: Metabolomics. 2024 May 11;20(3):54. doi: 10.1007/s11306-024-02127-w (PMC11088559; doi:10.1007/s11306-024-02127-w)
Supplement: Supplementary file 1 — Supplementary Material 1 [file 11306_2024_2127_MOESM1_ESM.docx]

**Characterizing poorly controlled type 2 diabetes using ^1^H-NMR metabolomics**

**Isabella J. Theron^1^, Shayne Mason^1^, Mari van Reenen^1^, Zinandré Stander ^1^, Léanie Kleynhans^2,3^, Katharina Ronacher^2,3,4^, Du Toit Loots^1*^**

^1^Human Metabolomics, Department of Biochemistry, Faculty of Natural and Agricultural Sciences, North-West University, Potchefstroom, South Africa; ^2^DSI-NRF Centre of Excellence for Biomedical Tuberculosis Research, South African Medical Research Council Centre for Tuberculosis Research, Division of Molecular Biology and Human Genetics, Department of Biomedical Sciences, Stellenbosch University, Cape Town, South Africa; ^3^Mater Research Institute - The University of Queensland, Translational Research Institute, Brisbane, Australia, The University of Queensland, Brisbane, Australia; ^4^Australian Infectious Diseases Research Centre, The University of Queensland, Brisbane, Australia.

**Orcid ID’s:**

Isabel Theron: <https://orcid.org/0000-0002-3514-6937>

Shayne Mason: <https://orcid.org/0000-0002-2945-5768>

Mari van Reenen: <https://orcid.org/0000-0002-5856-3258>

Zinandré Stander: <https://orcid.org/0000-0001-8281-5112>

L**é**anie Kleynhans: <https://orcid.org/0000-0003-1635-253X>

Katharina Ronacher: <https://orcid.org/0000-0003-2487-125X>

Du Toit Loots: <https://orcid.org/0000-0002-0339-6237>

**Email addresses:**

[Isabel.theron0@gmail.com](mailto:Isabel.theron0@gmail.com)

[nmr.nwu@gmail.com](mailto:nmr.nwu@gmail.com)

[van.reenen.mari@gmail.com](mailto:van.reenen.mari@gmail.com)

[Stander.Zinandre@mayo.edu](mailto:Stander.Zinandre@mayo.edu)

[leanie.kleynhans@mater.uq.edu.au](mailto:leanie.kleynhans@mater.uq.edu.au)

[katharina.ronacher@mater.uq.edu.au](mailto:katharina.ronacher@mater.uq.edu.au)

[dutoit.loots@nwu.ac.za](mailto:dutoit.loots@nwu.ac.za)

***Corresponding Author**

[dutoit.loots@nwu.ac.za](mailto:dutoit.loots@nwu.ac.za)

**Electronic supplementary material:**

**S1 Table 1** De-identified poorly controlled type 2 diabetes participants and their prescribed treatment

| **Participants** | | **Treatment** | | |
| --- | --- | --- | --- | --- |
|  |  | **Metformin** | **Insulin** | **Glimepiride** |
| **1** | | ✓ | - | - |
| **2** | | - | - | ✓ |
| **3** | | ✓ | ✓ | - |
| **4** | | ✓ | - | - |
| **5** | - | | - | - |
| **6** | | ✓ | - | - |
| **7** | | ✓ | ✓ | - |
| **8** | | ✓ | ✓ | - |
| **9** | | - | - | ✓ |
| **10** | | ✓ | ✓ | - |
| **11** | | ✓ | ✓ | - |
| **12** | | ✓ | - | - |
| **13** | | ✓ | ✓ | - |
| **14** | | ✓ | - | - |
| **15** | | ✓ | ✓ | ✓ |

*It is important to note that these participants weren’t necessarily on their prescribed treatment at the time of sample collection

^
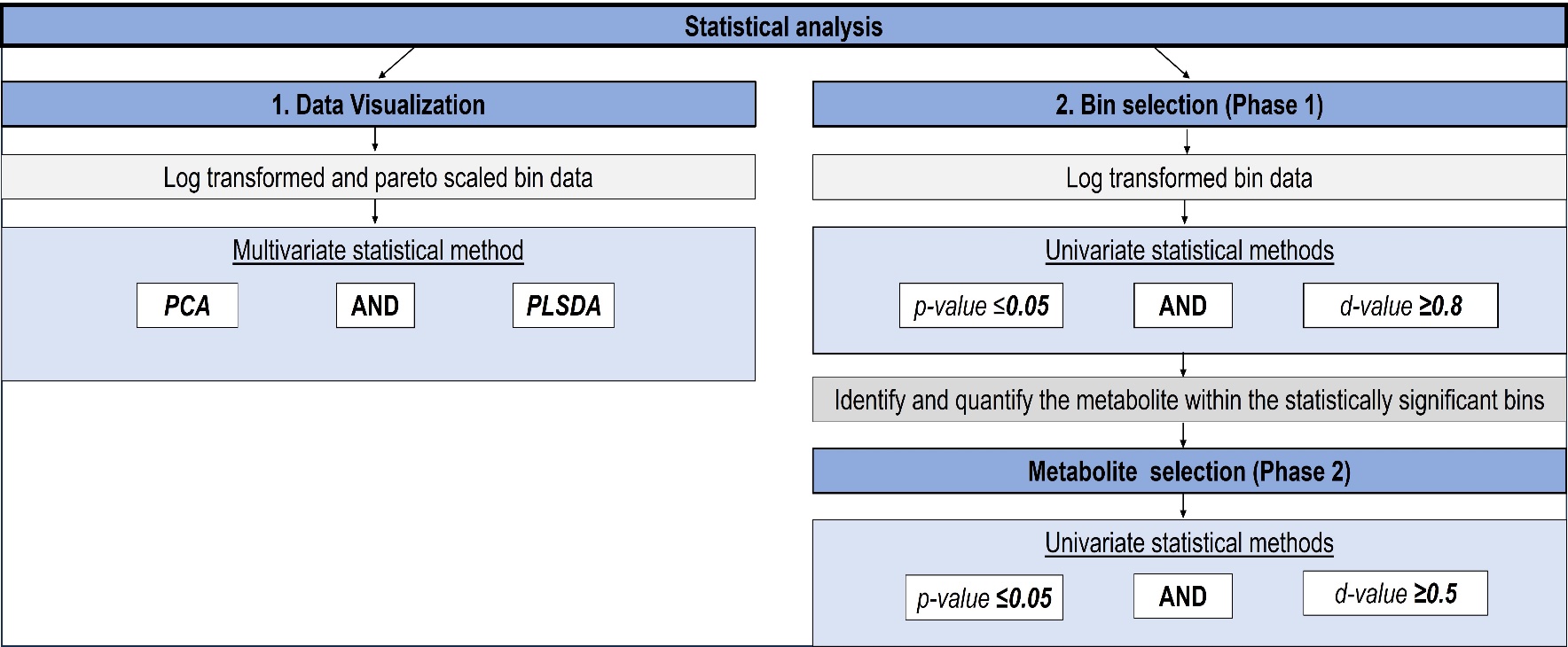
^

**S1 Fig 1** Schematic summary of the statistical analysis

*
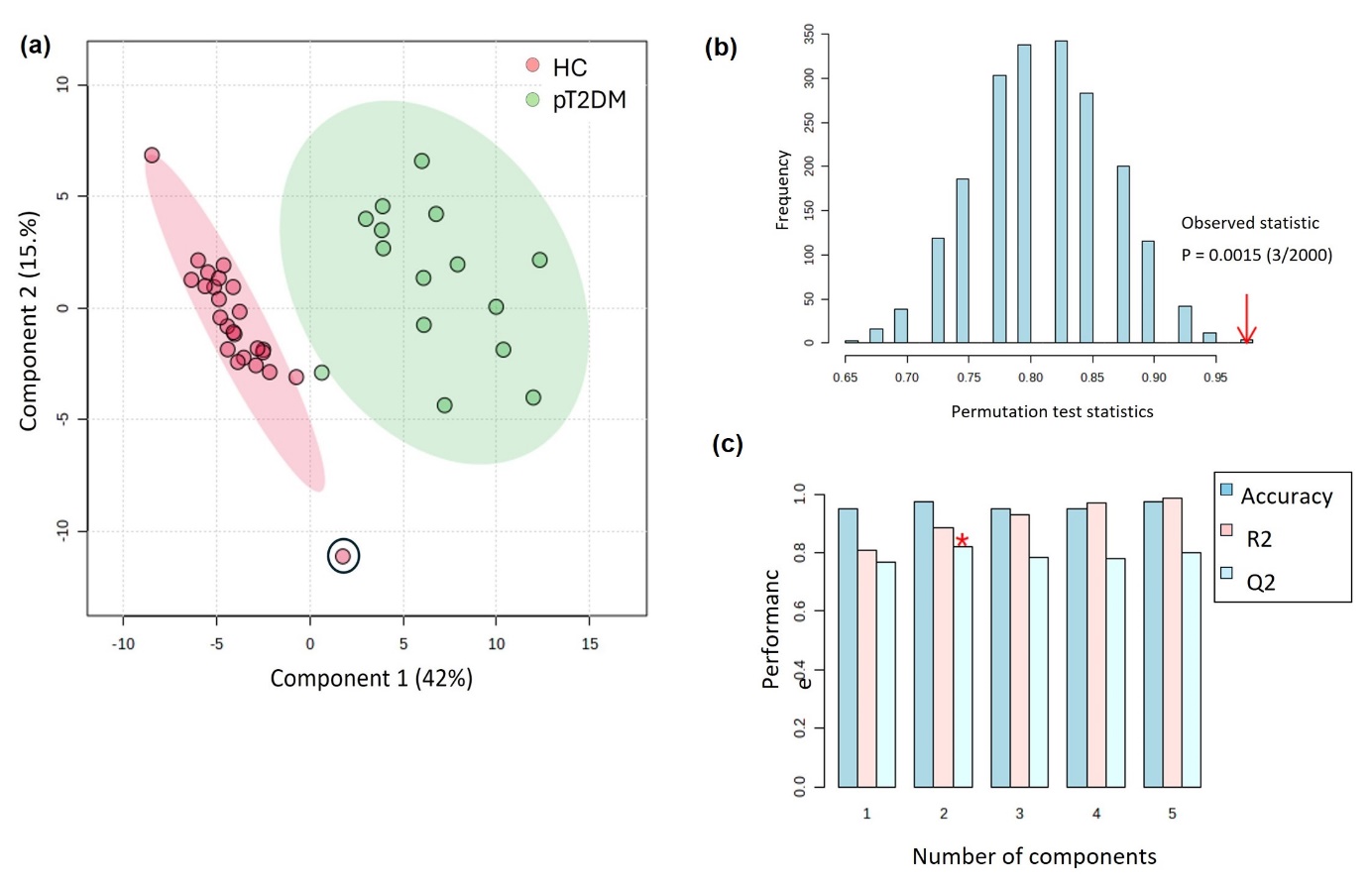
*The circled participant has been classified as an outlier.*

**S1 Fig 2** PLSDA, permutation test, and cross-validation **(a)** The PLSDA illustrates the supervised differentiation of the metabolome data of the 25 healthy control (HC) (pink circle) and 15 poorly controlled type 2 diabetes (pT2D) (green circle) participants’ urine metabolome profiles. **(b)** The permutation test on all samples: a p-value is ≤ 0.05, and a permutation of X2000, validates the stability of the model. **(c)** Using a leave-one-out cross validation of all samples, an R2, Q2 accuracy of > 0.8 was achieved.

**S1 Table 2** ^1^H-NMR metabolite assignments

| **Peak** | **Metabolites** | **Chemical shifts** | **Protons** | **Multiplicity** | **Moiety** |
| --- | --- | --- | --- | --- | --- |
| **1** | **α-Hydroxybutyric acid**  *(HMDB0000008)* | 0.89 | 3 | T | CH_3_ |
| **2** | **β-Hydroxybutyric acid**  *(HMDB0000011)* | 1.19 | 3 | D | CH_3_ |
| **3** | **β-Hydroxyisobutyric acid**  *(HMDB0000023)* | 1.07 | 3 | D | CH_3_ |
| **4** | **β-Hydroxyisovaleric acid**  (*HMDB0000754)* | 1.26 | 6 | S | (CH_3_)_2_ |
| **5** | **ρ-Hydroxyphenylacetate**  *(HMDB0060390)* | 1.81 | 1 | D | CH |
| **6** | **Acetoacetic acid**  *(HMDB0000060)* | 2.27 | 2 | S | CH_2_ |
| **7** | **Alanine**  *(HMDB0000161)* | 1.48 | 3 | D | CH_3_ |
| **8** | **Choline**  *(HMDB0000097)* | 3.20 | 9 | S | (CH_3_)_3_ |
| **9** | **Citric acid**  *(HMDB0000094)* | 2.54 | 1 | D | CH |
| **10** | **Dimethylglycine**  *(HMDB0000092)* | 2.92 | 6 | S | (CH_3_)_2_ |
| **11** | **Fumaric acid**  *(HMDB0000134)* | 6.53 | 2 | S | CH2 |
| **12** | **α-Glucose**  *(HMDB0000122)* | 5.25 | 1 | D | CH |
| **14** | **β-Glucose**  *(HMDB0000122)* | 4.66 | 1 | D | CH |
| **15** | **Isoleucine**  *(HMDB0000172)* | 1.02 | 3 | D | CH_3_ |
| **16** | **Lactic acid**  *(HMDB0000190)* | 1.33 | 3 | D | CH_3_ |
| **17** | **Leucine**  *(HMDB0000687)* | 0.97 | 6 | T | (CH_3_)_2_ |
| **18** | **Lysine**  *(HMDB0000182)* | 1.73 | 2 | T | CH_2_ |
| **19** | **Mannose**  *(HMDB0000169)* | 5.20 | 1 | D | CH |
| **20** | **Myo- inositol**  *(HMDB0000211)* | 4.07 | 1 | T | CH |
| **21** | **N-acetylglucosamine**  *(HMDB0000215)* | 2.07 | 3 | S | CH_3_ |
| **22** | **Pyroglutamate**  *(HMDB0000267)* | 4.18 | 1 | Q | CH |
| **23** | **Pyruvic acid**  *(HMDB0000243)* | 2.38 | 3 | S | CH_3_ |
| **24** | **Sucrose**  *(HMDB0000258)* | 5.42 | 1 | S | CH |
| **25** | **Tryptophan**  *(HMDB0000929)* | 7.70 | 1 | DD | CH |
| **26** | **Tyrosine**  (*HMDB0000158)* | 6.90 | 2 | DD | CH_2_ |
| **27** | **Valine**  *(HMDB0000883)* | 1.04 | 3 | D | CH_3_ |

HMDB The human metabolome database, s singlet, d duplet, t triplet, dd dubbel duplet, q quintet
